# A Cross-Sectional Study of Oral Lichen Planus Associated With Thyroid Diseases in East China

**DOI:** 10.3389/fendo.2019.00928

**Published:** 2020-01-24

**Authors:** Yunju Tang, Linjun Shi, Boren Jiang, Zengtong Zhou, Xuemin Shen

**Affiliations:** ^1^Department of Oral Mucosal Diseases, Shanghai Ninth People's Hospital, College of Stomatology, Shanghai Jiao Tong University School of Medicine, Shanghai, China; ^2^National Clinical Research Center for Oral Diseases, Shanghai Key Laboratory of Stomatology & Shanghai Research Institute of Stomatology, Shanghai, China; ^3^Department of Endocrinology and Metabolism, Shanghai Ninth People's Hospital, Shanghai Jiao Tong University School of Medicine, Shanghai, China

**Keywords:** oral lichen planus, thyroid disease, Hashimoto's thyroiditis, immunology, cross-sectional study

## Abstract

**Objective:** To investigate the prevalence of thyroid diseases in patients with oral lichen planus (OLP) and to explore the correlation between the two diseases.

**Methods:** A cross-sectional study was conducted to investigate the history of thyroid disease in 585 patients with oral lichen planus diagnosed in the Department of Oral Mucosal Diseases of the Ninth People's Hospital, Shanghai Jiaotong University, School of Medicine from June 2017 to April 2018 and in 10,441 normal people in an epidemiological survey conducted by endocrinology department of Ninth People's Hospitalin eastern China from 2014 to 2015. Personal medical history of thyroid disease was obtained through questionnaire and thyroid function was also tested.

**Results:** Of the 585 patients with OLP, 190 (32.48%) had thyroid disease (excluding coexistence of multiple thyroid diseases), 62 (32.6%) had thyroid nodules, and 71 (37.4%) had Hashimoto's thyroiditis. Hyperthyroidism was diagnosed in six patients (3.2%), hypothyroidism in seven patients (3.7%), and thyroid cancer in 11 patients (5.8%). The prevalence of Hashimoto's thyroiditis was significantly higher in patients with oral lichen planus than in the general population. The probability of thyroid disease was significantly higher in women with OLP than in men with OLP (*P* < 0.001).

**Conclusion:** OLP is associated with a high probability of developing thyroid disease, especially Hashimoto's thyroiditis. In the management of OLP patients, especially in female patients, thyroid disease must be screened.

## Introduction

Oral Lichen Planus (OLP) is a chronic inflammatory oral mucosal disease, which is common in middle-aged women (1). The clinical features of OLP is multifocal, usually bilateral affecting buccal mucosa, tongue, lips, gingiva, and white reticular patches with or without erosions and ulcerations. The histopathological features of OLP is Hyperkeratosis with basal cell degeneration, necrosis of basal, and parabasal keratinocytes, and a band-like predominantly lymphocytic infiltrate adjacent to basal cells (2). However, the etiology and pathogenesis of OLP are still enigmatic. Several studies indicated that OLP is closely related to some systemic diseases, such as diabetes mellitus (DM), hepatitis C and thyroid diseases (3, 4). Among these diseases, some scholars have shown that the immune system may play a critical role in the appearance of OLP in patients with type I DM (5). Thyroid diseases (TD) such as hyperthyroidism, thyroid cancer, and Hashimoto's thyroiditis (HT) have a diverse prevalence ranging from 0.5 to 1% in Europe, Japan, and the USA (6). However, in China, due to the huge population base, over 200 million people suffer from TD, where the prevalence of hypothyroidism and hyperthyroidism has been estimated to be 6.5 and 1.3%, respectively (7). In 2016, a study was conducted by Chinese Society of Endocrinology, revealing that more than 40% of the population suffered from TD (8). In the study of the relationship between OLP and TD, some scholars found that erosive OLP has been associated with anti-TPO autoantibodies (TPOAb) in thyroid patients, it may be useful to determine TPOAb levels of such patients to diagnose a possible undetected thyroid disorders and follow-up for malignancy (9). HT is a kind of TD with incidence from 0.4 to 2% in the general population (10). HT often causes thyroid dysfunction, especially hypothyroidism, and some HT patients require surgical treatment, which increases the burden of disease treatment. So, more attention should be paid to HT (11). Several studies in other populations have investigated the possible relationship between thyroid diseases and OLP (12–14). Only one literature from China reported the possible relationship between OLP and TD in northern Chinese population, and this study included <200 patients with oral lichen planus (15). However, the epidemiological characteristics of OLP with HT have never been discussed. Therefore, a larger sample size study was needed to enrich the knowledge of association between OLP and TD, especially HT.

## Materials and Methods

The present study was approved by the ethics committee of Shanghai Ninth People's Hospital (2016-201-T145), and written informed consent was obtained from all participants. A cross-sectional study was conducted to investigate 585 patients with oral lichen planus from June 2017 to April 2018. The personal medical history of thyroid disease was obtained by questionnaire, and thyroid function including thyroid peroxidase antibody (TPOAb), thyroglobulin (TGAb), thyroid-stimulating hormone (TSH), free triiodothyronine (FT3), free thyroxine (FT4) was also tested. Thyroid disease information of the 10,441 normal people as a control group came from an epidemiological survey conducted by endocrinology department of Shanghai Ninth People's Hospital in east China from 2014 to 2015. The gender, age, TPOAb, TGAb, TSH, FT3, FT4, and B-ultrasound results of these people are all from the epidemiological investigation (16).

### Inclusion Criteria

OLP patients who were diagnosed clinically and histologically according to the diagnostic criteria of AAOMP (17). Briefly, for the diagnosis of OLP, the clinical criteria included the presence of bilateral, more or less symmetric lesions; the lesions could be lace-like network of gray-white lines, plaque, atrophic, bullous, or erosive type. The histological criteria included the presence of a distinct band-like zone of cellular infiltration confined to the superficial part of the connective tissue, a sign of liquefaction degeneration in the basal cell layer and the absence of epithelial dysplasia. According to the guideline of diagnosis and treatment of thyroid diseases in China (18), the criteria for Hashimoto's thyroiditis (HT) included the positive detection of thyroid peroxidase antibody (TPOAb) and thyroglobulin (TGAb), with or without local and systemic manifestations including dysphonia, dyspnea, and constipation (19). For hypothyroidism, it included the higher value of thyroid-stimulating hormone (TSH), the lower value of total thyroxine (TT4), and free thyroxine (FT4). For hyperthyroidism, it included the lower value of TSH, the higher value of TT4, FT4, total triiodothyronine (TT3), and free triiodothyronine (FT3), with or without clinical symptoms and signs of clinical hypermetabolism, such as increased heart rate. For thyroid nodules, it included one or more lump in the thyroid gland detected by B ultrasound.

### Exclusion Criteria

Patients under 18 years old or who were pregnant; patients with a history of action capability restricting or life-threatening systemic diseases like uremia, or other autoimmune diseases which seriously affects the quality of life, such as psoriasis, vitiligo, Behcet disease, or bullous diseases.

### Data Collection

The demographic and clinical information of the participants was recorded including age, gender, the score of clinical manifestation, medication history, and general condition. No more thyroid examination including B-ultrasound was performed for participants with diagnosed thyroid diseases. On the contrary, if the participants had not been diagnosed with any thyroid disease in the past, these participants were instructed to undergo a thyroid examination including thyroid function (FT3, FT4, and TSH), thyroid related antibodies (TPOAb and TGAb). These indicators were detected by chemiluminescence immunoassay (CLIA). And, these participants also received a B-ultrasound of the thyroid gland (20). Results of the thyroid examination were determined by two independent endocrinologists of Shanghai Ninth People's Hospital to confirm the diagnosis of HT, hypothyroidism, thyroid nodule, and hyperthyroidism accordingly.

### Statistical Analysis

The data were processed by SPSS 23.0 software package. We calculated the odds ratio (OR) with 95% confidence intervals (CI) for associations between OLP and control group in different types of thyroid diseases. Comparison of parameters of age and OLP score between the OLP and control group was performed by *T*-test. Comparison of parameters of Gender, Smoking history and Clinical type between the OLP and control group was performed by χ^2^ test. The difference was statistically significant with *P* < 0.05.

## Results

**Figure 1:**
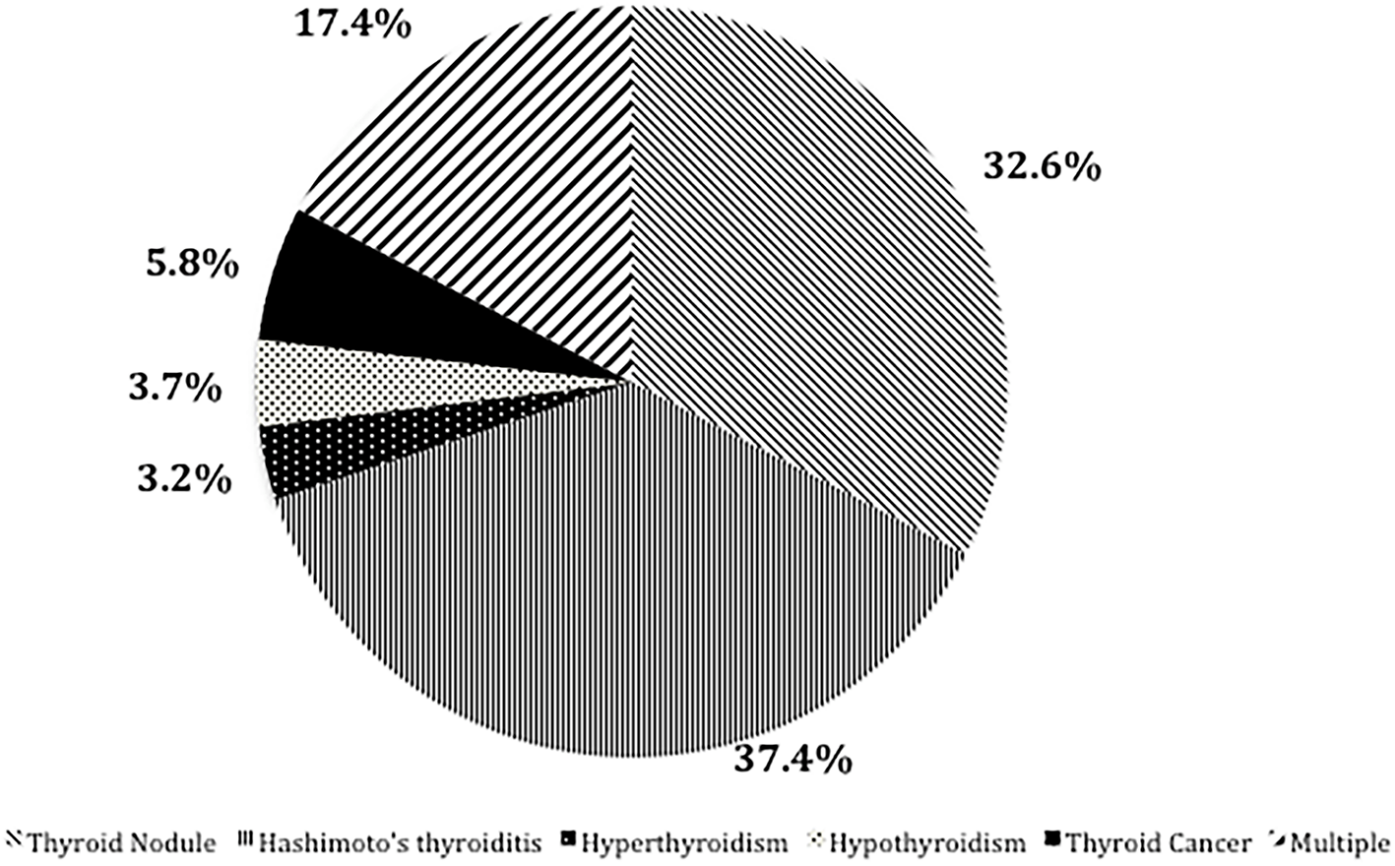
Rate of different thyroid diseases (excluding coexistence of multiple thyroid diseases) in OLP patients with TD.

The proportion of male and smoking history in OLP patients with TD was significantly lower than that in patients without TD (Table 1). Rate of different thyroid diseases (excluding coexistence of multiple thyroid diseases) in OLP patients with TD (Figure 1, Table 2). The proportion of female OLP patients was higher than that in control group (Table 3). HT ranked first in the OLP-associated TD, followed by thyroid nodules. The prevalence of HT and Multiple in OLP patients was significantly higher than that in control group. The prevalence of Thyroid nodule and Hypothyroidism in OLP patients was significantly lower than that in control group (Table 4). Among female, the prevalence of HT in OLP patients was significantly higher than that in control group, but there was no difference among male (Table 5). Considering HT was the most common type of TD in OLP patients, so we specially analyzed the situation of OLP with HT. Similar to the results in Table 1, the two groups of patients also had obvious differences in gender and smoking history (Table 6).

**Table 1:** Difference of demographics information and clinical information between OLP with TD and OLP without TD.

	**OLP with TD**	**OLP without TD**	***P*-value**
Number	190	395	
**GENDER**			
Male (%)	23 (12.1)	121 (30.6)	*P <* 0.001
Female (%)	167 (87.9)	274 (69.4)	
Age	52.85 ± 13.15	52.72 ± 13.71	*P* > 0.05
Smoking history (%)	26 (13.7)	126 (31.9)	*P <* 0.001
**CLINICAL TYPE**			
Erosive (%)	26 (13.7)	44 (11.1)	*P* > 0.05
Non-erosive (%)	164 (86.3)	351 (88.9)	
OLP score	4.70 ± 2.37	4.56 ± 2.50	*P* > 0.05

**Table 2:** Rate of different thyroid diseases (excluding coexistence of multiple thyroid diseases) in OLP patients with TD.

**Different thyroid diseases among OLP patients**	**Number**	**Rate of different thyroid diseases in OLP patients with TD, %**
Thyroid nodule	62^a^	32.6
Hashimoto's thyroiditis	71	37.4
Hyperthyroidism	6	3.2
Hypothyroidism	7	3.7
Thyroid cancer	11	5.8
Multiple	33	17.4
Total	190	100

a *Sixty-two patients with thyroid nodules in OLP patients were from 212 patients who received a B-ultrasound of the thyroid gland*.

**Table 3:** Demographics information between OLP patients and control group.

	**OLP**	**control group**	***P*-value**
Number	585	10,441	
**GENDER**			
Male (%)	144 (24.6)	4,310 (41.3)	*P <* 0.05
Female (%)	441 (75.4)	6,131 (58.7)	
Age	52.80 ± 13.52	53.50 ± 13.05	*P* > 0.05

**Table 4:** Number and rate of different thyroid diseases (excluding coexistence of multiple thyroid diseases) in 585 OLP patients and control group.

**Different thyroid diseases among OLP patients**	**Number**	**Rate of different thyroid diseases in OLP patients, %**	**Number**	**Population rate of control group, %**	***P*-value**	**OR [95% confidence intervals (CI)]**
Thyroid nodule	62^a^	10.6	5,183	49.6	*P <* 0.05	0.120 (0.092, 0.157)
Hashimoto's thyroiditis	71	12.1	638	6.1	*P <* 0.05	2.122 (1.635, 2.755)
Hyperthyroidism	6	1.0	48	0.5	*P* > 0.05	2.244 (0.956, 5.264)
Hypothyroidism	7	1.2	430	4.1	*P <* 0.05	0.282 (0.133, 0.598)
Thyroid cancer	11	1.9	117	1.1	*P* > 0.05	1.691 (0.906, 3.155)
Multiple	33	5.6	193	1.8	*P <* 0.05	3.174 (2.173, 4.637)
Other thyroid diseases	NA	NA	22	0.2	NA	NA

a *Sixty-two patients with thyroid nodules in OLP patients were from 212 patients who received a B-ultrasound of the thyroid gland*.

**Table 5:** The prevalence of HT in OLP patients and the normal people was compared between male and female, respectively.

**Gender**	**Group**	**Number and rate of HT in OLP patients**	**Number and rate of HT in normal people**	***P*-value**
Male	HT	8 (5.6%)	134 (3.1%)	*P* > 0.05
	Total	144	4,310	
Female	HT	63 (14.3%)	504 (8.2%)	*P* < 0.05
	Total	441	6,131	

**Table 6:** Demographics information and clinical information from the OLP with HT and OLP without TD.

	**OLP with HT**	**OLP without TD**	***P*-value**
Number	97	395	
**GENDER**			
Male (%)	10 (10.3)	121 (30.6)	*P* < 0.001
Female (%)	87 (89.7)	274 (69.4)	
Age	50.77 ± 13.44	52.72 ± 13.71	*P* > 0.05
Smoking history (%)	11 (11.3)	126 (31.9)	*P* < 0.001
**CLINICAL TYPE**			
Erosive (%)	13 (13.4)	44 (11.1)	*P* > 0.05
Non-erosive (%)	84 (86.6)	351 (88.9)	
OLP score	4.71 ± 2.36	4.56 ± 2.50	*P* > 0.05

## Discussion

Although the pathogenesis of OLP is still unclear, recently, more and more studies have shown that OLP is related to systemic diseases such as autoimmune diseases, endocrine diseases, and metabolic diseases (1, 21). Researchers in China and other countries have paid much attention to thyroid diseases among all kind of systemic diseases, but most of them have few cases and fewer thyroid diseases (22). However, study from China with large sample still lacked evidence of the relationship between OLP and thyroid diseases, hence the present study was designed to enrich the knowledge. The majority of OLP patients we collected were middle-aged and elderly female, which was consistent with the characteristics of OLP in the past study (23). Among the systemic diseases associated with OLP, more attention to TD was paid. The incidence of TD is higher than the results of other researchers but not significantly higher than that of the general population (*p* > 0.05). In the present study, female OLP patients older than 40 years old were more likely to suffer from TD probably because both OLP and thyroid diseases were prevalent among middle-aged and elderly females.

This study expanded the sample size and focused on the classification of thyroid diseases associated with OLP patients. Among OLP with TD, the present study found that Hashimoto thyroiditis and Thyroid nodule were prevalent.

In the present manuscript, although HT is the most common thyroid disease among OLP patients, OLP with HT has no particularity in OLP with TD in terms of demographic information and clinical manifestations. Based on its own research and the results of literature review, it screened out the more possible mechanisms from the respective pathogenesis of the two diseases and discussed them. There are roughly the following theories: (1) HT is mostly originated from OLP. Some scholars speculated that the changes in hormone levels and thyroid autoantibodies caused by HT may lead to changes in oral local keratinocytes, thus triggering OLP (24). (2) The peripheral blood and oral lesions of OLP patients showed T lymphocyte distribution different from that of healthy people, with abnormal increase of cytokines TNF-α and IFN-γ (25, 26). The peripheral blood of HT patients also showed similar phenomenon (27, 28), so this immunological change may be the common pathogenesis of both, and OLP and HT may also be two clinical manifestations of a certain syndrome. (3) In terms of genetic factors, there were also relevant researches on the susceptibility genes of OLP and HT. The coexistence of the two susceptibility genes may also be the cause of the two diseases, but the relationship between the two susceptibility genes has not yet been determined (29). Since the pathogenesis of HT and OLP was similar, both of which were T cell mediated local immune responses, so the relationship between the two needed more attention, and cellular immunity might be a better research direction. Since it is impossible to distinguish whether the results are caused by OLP alone or HT or by a combination of two diseases, although the pathogenesis of OLP and HT has many similarities, it is still necessary to study the pathogenesis of OLP and HT. And more attention should be paid to the primary and secondary diseases, which is the main obstacle for our future research.

The thyroid disease information of some patients with OLP came from the medical history, and the information of the other patients came from the examinations that were done at that time. Although the prevalence of thyroid nodules was significantly different between the two groups, 62 patients with thyroid nodules in OLP patients were from 212 patients who received B-ultrasound of the thyroid gland, the prevalence of thyroid nodules in OLP patients was not representative. Thyroid nodules ranked second in the prevalence of thyroid disease associated with OLP patients. Thyroid nodules are common, being detected in up to 65% of the general population. Most thyroid nodules are benign, clinically insignificant, and safely managed with a surveillance program. However, this nodule that harbor a clinically significant cancer (≈10%) (30). Thyroid cancer is one of the cancers with lower mortality rate, but it ranks fourth in the incidence of women (31). Therefore, it is necessary to discover thyroid cancer in advance. Among patients with OLP, 11 patients developed thyroid cancer after OLP. The thyroid cancer in these patients was caused by malignant transformation of thyroiditis or thyroid nodules, often accompanied by other thyroid diseases. This phenomenon deserves our close attention. Although the mortality rate of thyroid cancer is not high, the incidence rate of thyroid cancer is increasing, including malignant transformation of various thyroid diseases and primary thyroid cancer, so the economic burden of the disease is also increasing (32). It is still too early to use OLP as an early warning indicator for thyroid cancer. However, examination of serum thyroid function and thyroid B ultrasound examination in OLP patients is necessary for the diagnosis of thyroid disease in order to find thyroid cancer at an early stage, especially for the elderly and women.

The limitation in this study is that the data of the control group are historical data and there is no comparison between the data at the same time.

## Conclusion

To sum up, the present study showed that OLP was closely related to HT through a larger sample size. The possible mechanism behind this correlation is still enigmatic and deserves further research. Middle-aged women with OLP needed to undergo thyroid blood tests and B ultrasound to screen for thyroid diseases.

## Data Availability Statement

All datasets generated for this study are included in the article/supplementary material.

## Ethics Statement

The studies involving human participants were reviewed and approved by the ethics committee of Shanghai Ninth People's Hospital (2016-201-T145). The patients/participants provided their written informed consent to participate in this study.

## Author Contributions

YT and XS developed the project and implementation scheme. YT, BJ, and XS recruited and collected clinical cases. YT performed the statistical analysis of relevant data. YT and LS participated in the writing and modification of the article. XS and ZZ critically revised the manuscript.

### Conflict of Interest

The authors declare that the research was conducted in the absence of any commercial or financial relationships that could be construed as a potential conflict of interest.
